# Optimal design and experimental research on the spiral groove wheel fertilizer apparatus

**DOI:** 10.1038/s41598-024-51236-y

**Published:** 2024-01-04

**Authors:** Fujun Wen, Honghai Wang, Lai Zhou, Qingchuang Zhu

**Affiliations:** 1 Guangzhou Panyu Polytechnic, Guangzhou, 511483 People’s Republic of China; 2 Mingjiang Town People’s Government of Ningming County, Chongzuo City, 532503 Guangxi People’s Republic of China

**Keywords:** Engineering, Mechanical engineering

## Abstract

As the "core" of fertilizer machinery, the fertilizer apparatus has a significant impact on the effect of fertilization operation. At present, the straight-grooves type external groove wheel fertilizer apparatus is widely used, which has the problem that the fertilizer flow fluctuates periodically and is not conducive to precision fertilization. Therefore a kind of the spiral groove wheel fertilizer apparatus is proposed in the paper, and the designed fertilizer apparatus is experimented and optimized by using Design-Expert Software 10 and a self-made fertilizer experiment bench. Taking the fertilization amount and the variation coefficient of fertilization amount as the experiment index, the interaction effects of the groove radius, spiral rising angle, groove wheel rotation speed, and fertilizer tongue inclination of the spiral groove wheel type fertilizer wheel on the fertilization performance are analyzed. The quadratic regression models of multiple factors of the fertilization amount and the variation coefficient of fertilization amount are established. Through optimization and experiment validation, the optimal combination of parameters is determined as follows: the groove radius is 13.5 mm, the spiral rising angle is 62°, the groove wheel rotation speed is 29.4 r/min, the fertilizer tongue inclination is 5°, the fertilization amount is 17.810 g and the variation coefficient of fertilization amount is 5.60%.

## Introduction

Rational and scientific fertilization is one of the main ways to reduce agricultural pollution, reduce agricultural production costs, promote agricultural production, and increase farmers' income^1^. Fertilization methods mainly include artificial fertilization and mechanical fertilization. Traditional artificial fertilization has disadvantages such as high cost, high labor intensity, and uneven application. The use of mechanical fertilization can reduce production costs, reduce labor intensity and improve fertilization uniformity. Therefore, mechanical fertilization is the main method of fertilization at present^2,3^.

Due to the differences in topography, farming methods, crop types and environmental climate, the fertilizer applicators used are different, and the matching fertilizer dispensers on the fertilization machines are also different. At present, there are nearly 20 types of fertilizer apparatuses used in agricultural production. The commonly used fertilizer apparatuses include disc rotary type fertilizer apparatus, external groove wheel fertilizer apparatus, guide plate rotary disk type fertilizer apparatus, horizontal star wheel type fertilizer apparatus, etcetera^4^. The large-scale fertilizer apparatus is mainly equipped in the seeder, and the sowing operation and fertilization operation are carried out at the same time, for example, the single-vibration large-groove wheel fertilizer apparatus and the double-vibration square-shaft fertilizer apparatus of the British potato planter, and the nail-wheel fertilizer apparatus used in Denmark and other European countries, these fertilizer apparatuses have uniform fertilization and high productivity^5^. Some countries have a wide crop area and relatively equal terrain, so special fertilizer dispensers are used for spreading before soil preparation, intertillage fertilization, soil improvement, etc. It mainly uses the high-speed rotating fertilizing disc to achieve the purpose of precise fertilization, the working mechanism of the centrifugal fertilizer spreader is to study the movement of fertilizer particles on the disc, and its analysis model can predict the trajectory of a single particle on the disc^6^. Van Liedekerke et al.^7^ used the discrete element method to simulate and analyze the trajectory of a single fertilizer particle on the rotating disc model of the fertilizer spreader. The simulation results of EDEM were compared with the actual fertilization test, and the simulation results were in good agreement with the test results. At present, the disc-type variable fertilizer spreaders have been produced as commodities, such as the MDS series disc-type fertilizer spreaders produced by the French Kuhn company, the DPX Prima fertilizer spreaders produced by the French Greiger-Besson company, etcetera^8,9^. In recent years, with the rapid development of fertilizer technology and precision agriculture, fertilizers with good particle mobility such as compound fertilizer, compound fertilizer, and slow-release fertilizer are increasingly used. Due to the characteristics of simple structure, convenient operation, good versatility and low cost, the external groove wheel fertilizer device is one of the most widely used fertilizer devices^10,11^.

As the "core" of fertilization machinery, the fertilizer apparatus has a significant impact on the effect of fertilization operation. The performance of fertilizer apparatus directly affects the amount, accuracy and stability of the fertilization process, that is, whether it meets the requirements of agricultural production. However, since the traditional external groove wheel fertilizer apparatus adopts the straight-grooves type, the fertilizer is discharged in the form of self-flow at the fertilizer discharge port. When the groove wheel turns to the groove, more fertilizer is discharged, while less fertilizer is discharged at the tooth ridge, and the fertilizer flow is periodically pulsating, especially at low speeds, which affects the uniformity of fertilization and is not conducive to precision^12–14^. Because of the above problems, this paper proposes a spiral groove wheel fertilizer apparatus, which has higher precision and more stable fertilizer discharge control. This product has certain practical significance and theoretical value.

Since 2020, with the rapid development of computers and other related technologies, numerical simulation has also been developed very quickly, especially the numerical simulation of particle motion is more and more attention from scholars. EDEM is an excellent discrete element simulation software for particles, and there are scholars at home and abroad who use EDEM to research the physical properties of particles^15^. In terms of soil, some scholars have used discrete element simulation techniques to study the influence of the surface morphology and the cut angle of the bulldozer plate on the dynamic behavior of the soil. By introducing parallel constraints to characterize the cohesive interaction between soil particles, a nonlinear mechanical model of the soil particle contact is established, and the perturbation behavior generated between the relevant components and soil is analyzed^16^. High-speed cameras and EDEM simulation were used to study the rolling friction characteristics of corn seeds, and the deviation between the test results and the simulation results was small, which means that the simulation was able to simulate the actual situation^17^. EDEM was used to simulate the flow accumulation behavior of particles under different physical conditions, and physical tests showed that the measured results were in good agreement with the numerical simulation^18^. The effect of the rolling friction coefficient on stacking characteristics was simulated on the product of Japonica rice after hulling, and the secondary simulation parameters were calibrated. The simulation results coincide with the experimental results, which indicate that the simulation of the stacking process by the discrete-element method can provide an effective solution for the determination of physical parameters not easily measurable in the bulk particles^19^.

### Structure of the spiral groove wheel fertilizer apparatus

**Figure 1:**
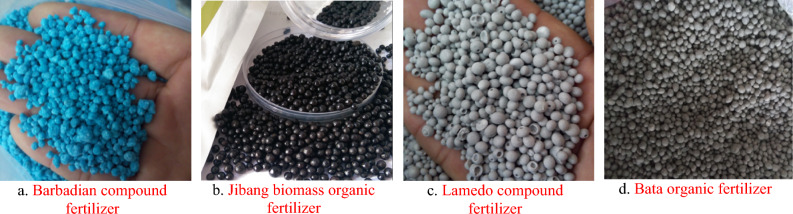
Fertilizer granules.

There are numerous classifications of fertilizers, which can be divided into organic fertilizers and chemical fertilizers according to their composition, and powder fertilizers, granular fertilizers and liquid fertilizers according to their physical form. Granular fertilizers are widely used due to their physical properties, ease of transportation and storage, ease of application, and slow release. In orchards, the commonly used granular fertilizers are compound mixed fertilizers, and the fertilizer apparatus in paper is applied to granular fertilizers. Several kinds of granular fertilizers are shown in Figure 1, among which Jibang biomass organic fertilizer is the main research object due to its more uniform fertilizer, regular shape, and wider application.

**Figure 2:**
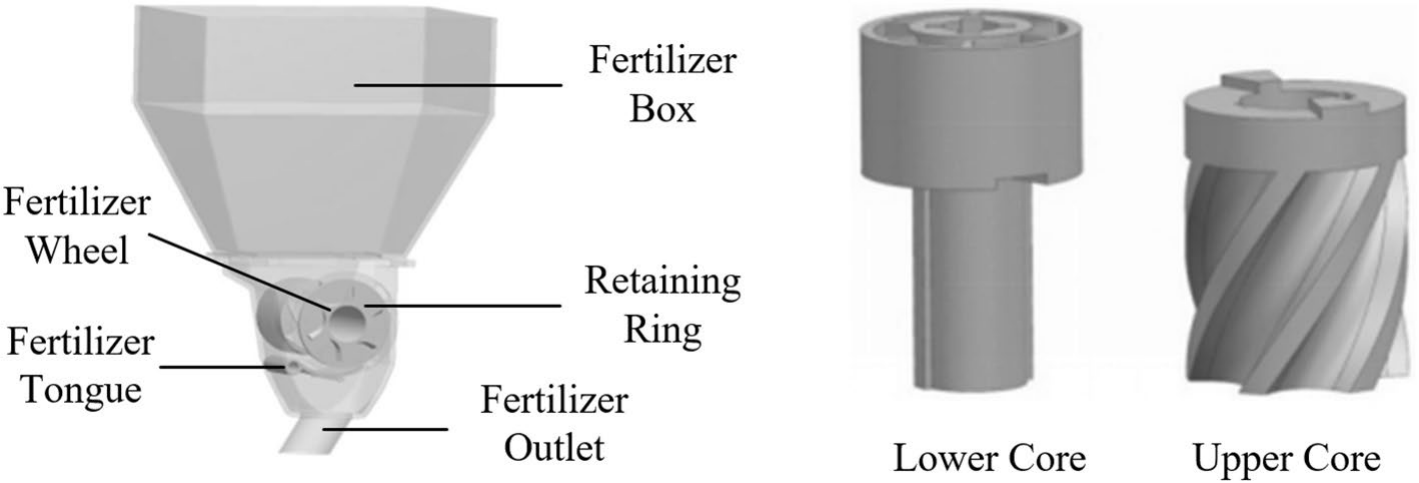
The structure of the fertilizer apparatus and spiral groove wheel.

As shown in Figure 2, the structure of the spiral groove wheel fertilizer apparatus is mainly composed of a fertilizer wheel, retaining ring, fertilizer box, fertilizer tongue, and fertilizer mouth. In order to improve the pulsation characteristics of the ordinary external groove wheel fertilizer apparatus and the accuracy of fertilization, the fertilizer wheel adopts the spiral groove wheel with an optimized design and the outer end of the fertilizer tongue has a certain inclination in the fertilizer apparatus proposed^10,20–23^. The spiral groove wheel designed is composed of the upper core (the spiral groove part) and the lower core (the cylinder part). The advantages of using this structure are as follows: (1) It is easy to install and debug. (2) The lower core can be interchanged with different spiral grooves of equal diameter, which means that if different groove radii, different spiral rising angles and different number of groove wheels are required, only the upper core needs to be replaced to save materials and costs.

The retaining ring corresponding to the spiral groove wheel is also designed. The retaining ring can move along the groove wheel, so as to change the working length of the groove wheel and adjust the fertilization amount. In addition, the fertilization amount can also be adjusted by rotating speed. When the fertilizer wheel rotates, the spiral groove wheel and the retaining ring rotate together with the shaft. The flower-shaped retaining ring prevents fertilizer leakage and reduces the wear of the spiral groove wheel. The retainer is fixed on the housing to prevent the axial movement of the flower-shaped retaining ring. The opening of the fertilizer tongue can be adjusted to suit fertilizers of different particle sizes. If the opening is too large, some fertilizers may flow automatically to affect the uniformity and stability of fertilization. If the opening is too small, the fertilizer particle breakage rate is large, which may also affect the uniformity and stability of fertilization.

When the spiral groove wheel fertilizer apparatus is fertilizing, the fertilizer particles fill the groove of the spiral groove wheel under the action of gravity. The fertilizer in the groove, which is called the active layer, is forced out with the rotation of the spiral groove wheel. The fertilizer outside the groove wheel, which is called the passive layer, is driven to discharge under the action of the pulling force of the outer circle of the groove wheel and the friction between the fertilizers. The fertilizers in the active layer and passive layer are squeezed into the fertilizer tongue and then are applied into the soil through the fertilizer pipe from the fertilizer outlet^24^.

The fertilization amount of the spiral groove wheel fertilizer apparatus, i.e. the fertilization amount of each rotation of the groove wheel, can be given as:

1 $$q = \pi DL\gamma \left( {\frac{{\alpha_{0} S}}{t} + \lambda } \right)$$

where *q* is the fertilization amount of the spiral groove wheel fertilizer apparatus, g/r; *D* is the groove wheel diameter, mm; *L* is the effective working length of the groove wheel, mm; *γ* is fertilizer particle density^25^, 1.32×10^−3^ g/mm^3^; α_0_ is the fertilizer filling coefficient in the groove; *S* is the cross-sectional area of a single groove, mm^SPS:refid::bib22^; *t* is the groove pitch, *t*=*πd/z*, and *z* is the groove number; *λ* is the characteristic parameter of the driving layer.

**Figure 3:**
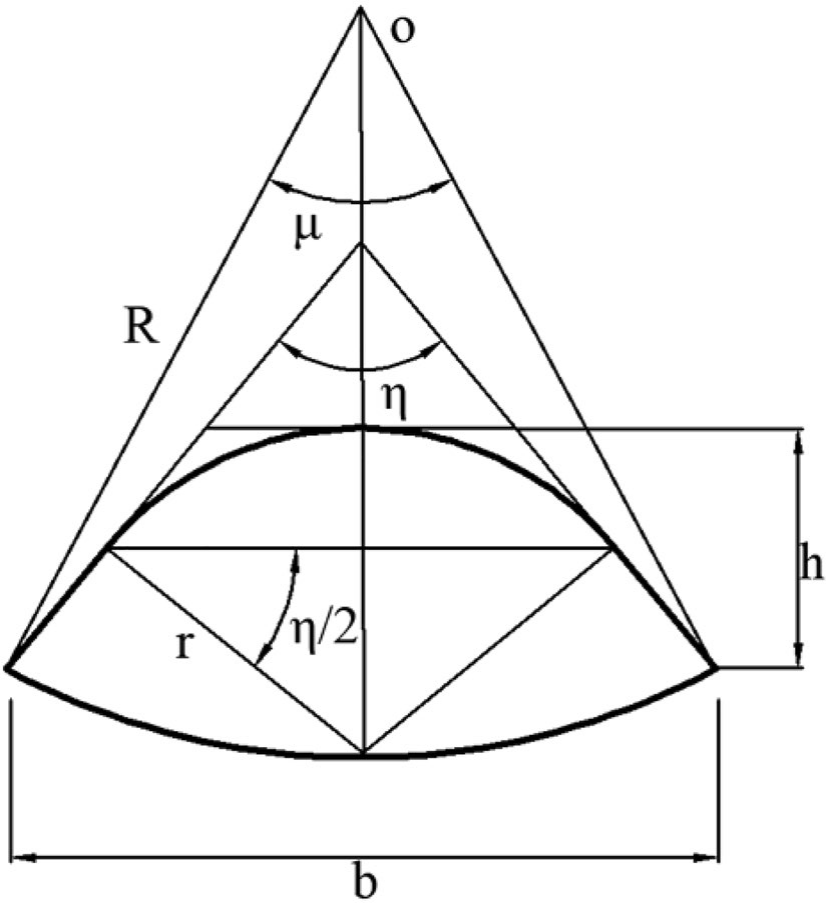
The structure of the fertilizer apparatus and spiral groove wheel.

*S* can be obtained from the geometry of the groove wheel. As shown in Figure 3, the cross-sectional area of the groove can be expressed as:

2 $$\begin{aligned} S = & \frac{{(180^{ \circ } - \eta )\pi r^{2} + \mu \pi R^{2} }}{{360^{ \circ } }} + r^{2} \left( {\cos \frac{\eta }{2}\sin \frac{\eta }{2} - \cos \frac{\eta }{2} - \cos \frac{\mu }{2}\sin \frac{\eta }{2} + \frac{{h\cos \frac{\eta }{2}}}{r}} \right) \\ + \frac{b}{2}\left( {r\sin \frac{\eta }{2} - r + h - R\cos \frac{\mu }{2}} \right) \\ \end{aligned}$$

where *η* is the included angle of two tangent lines of the groove edge; *r* is the groove radius, mm; *μ* is the central angle of the groove wheel corresponding to the groove, °; *R* is the groove wheel radius, mm; *h* is the depth of the groove, mm; *b* is the width of the groove, mm.

### Parameter design of the spiral groove wheel fertilizer apparatus

#### Parameter design of the spiral groove wheel

**Figure 4:**
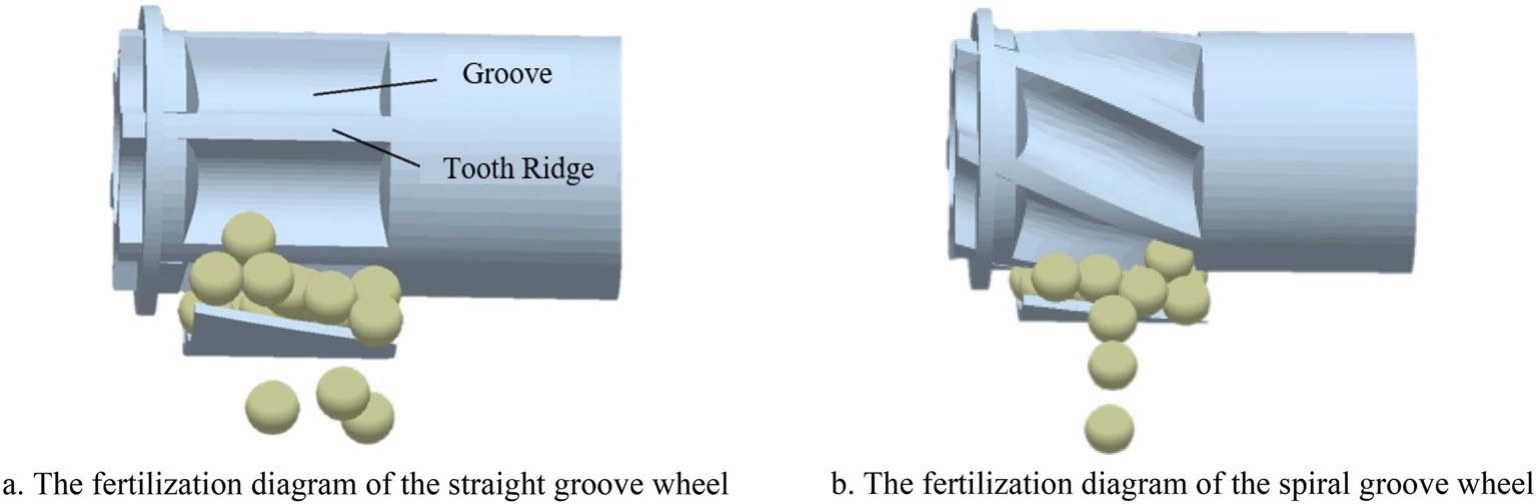
The fertilization diagram of different types of groove wheels.

The traditional external groove wheel fertilizer apparatus is mainly straight groove type (the spiral rising angle is 90°), and the fertilizer is discharged at the fertilizer outlet in the form of gravity flow. When the groove wheel turns to the groove, the fertilizer in the whole groove is discharged at the same time. At this time, more fertilizer is discharged, which is called the "crest". While less fertilizer is discharged at the tooth ridge, which is called the "trough". As shown in Figure 4a, assuming that *t* is the time required for the groove wheel to rotate through the two grooves in the figure, the cycle of "crests and troughs" is carried out twice within *t* time. At this time, the fertilizer flow shows a periodic pulsation phenomenon with a pulse frequency of 2/*t*, which affects the uniformity of fertilization and is not conducive to precision fertilization, especially at low speed and small grooves. To solve the above problems, the straight groove wheel is changed to a spiral groove wheel in paper, as shown in Figure 4b. Since the groove wheel is spiral, the fertilizer is continuously discharged at the fertilizer outlet, which effectively reduces the "crests and troughs" in the pulsation phenomenon and significantly reduces the difference between the maximum and minimum values, thus improving the uniformity of fertilization.

The diameter of the groove wheel has an impact on the fertilization performance of the fertilizer apparatus. In order to be interchangeable with the purchased external groove wheel fertilizer apparatus, the groove wheel diameter *D* is 61.6 mm and the effective working length of the groove wheel *L* is 63 mm. The groove radius also affects the uniformity and amount of fertilization. Considering the actual requirements of the groove radius, groove number, and fertilization amount in the orchard, the number of grooves *Z* is selected as 6 and the groove radius *r* is set to 9 – 14mm.

**Figure 5:**
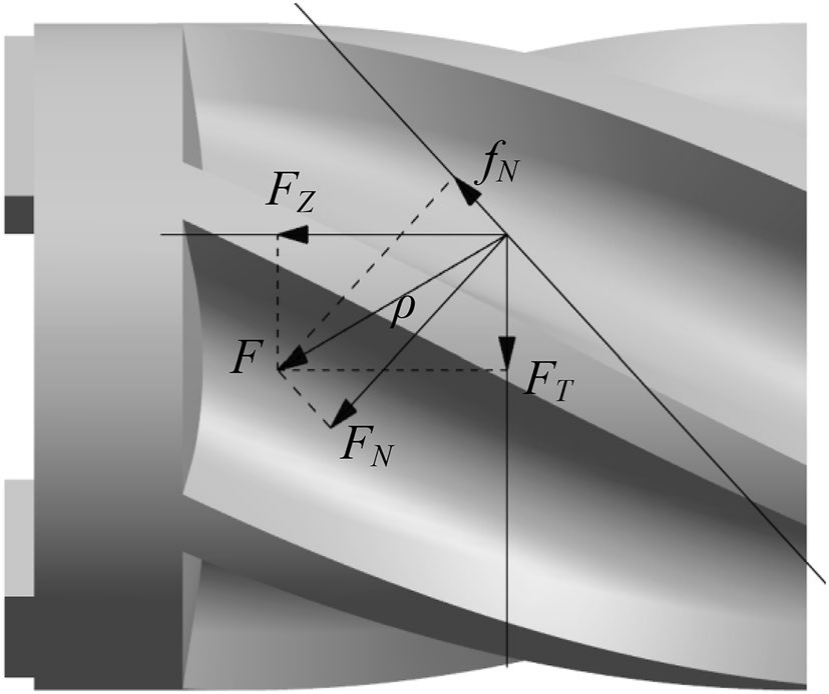
The horizontal force diagram of fertilizer particles.

In the fertilization process of the spiral groove wheel, the fertilizer moves in axial and radial directions under the action of the rotating spiral groove wheel. To carry out the dynamic analysis of fertilizer particles in the spiral groove wheel, assuming no relative slip between particles and ignoring the effect of surface roughness of the spiral groove wheel. A single fertilizer particle at distance* r* from the axis is selected and simplified to particle A as the research object^26,27^. When the spiral rising angle *α* is expanded and the spiral line is represented by an oblique line, the force diagram of fertilizer particles in the horizontal plane is shown in Figure 5.

As shown in Figure 5, *F*_N_ is the normal thrust from the spiral groove on the fertilizer particles; *f*_N_ is the tangential friction generated on the contact surface between the spiral groove wheel and fertilizer particles. *F* is the resultant force of *F*_N_ and *f*_N_; *ρ* is the friction angle of fertilizer particles after ignoring the roughness of the spiral groove wheel. Thus, the resultant force *F* can be decomposed into the circular force and axial force of fertilizer particles:

3 $$\left\{ \begin{gathered} F_{T} = F\sin (\alpha + \rho ) \hfill \\ F_{Z} = F\cos (\alpha + \rho ) \hfill \\ \end{gathered} \right.$$

where *F*_T_ is the circular force of fertilizer particles; *F*_Z_ is the axial force of fertilizer particles; *F*=*F*_N_/cos*ρ*.

**Figure 6:**
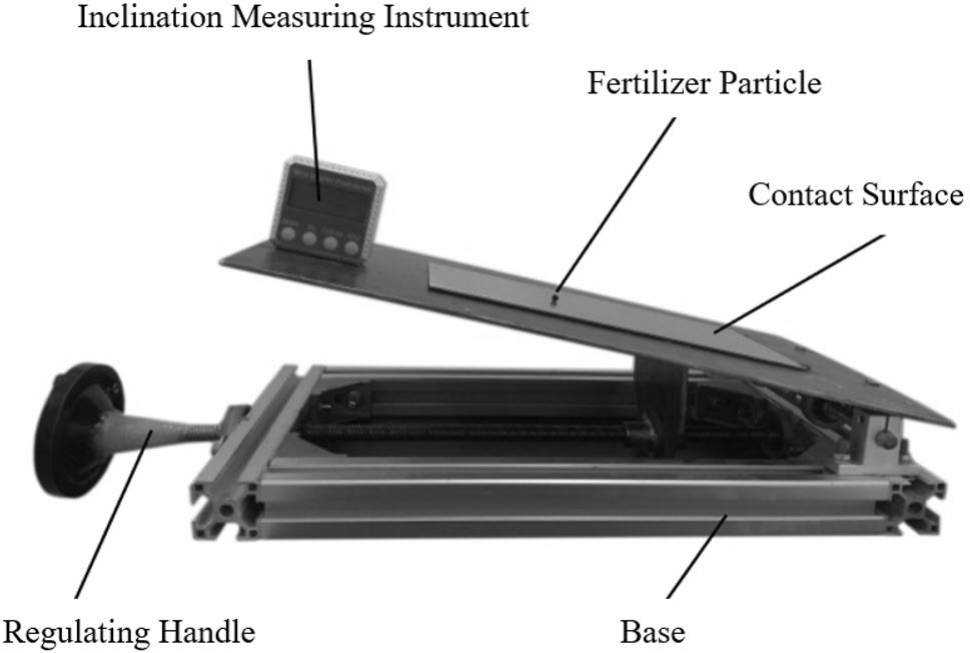
The self-made friction coefficient measuring instrument.

**Figure 7:**
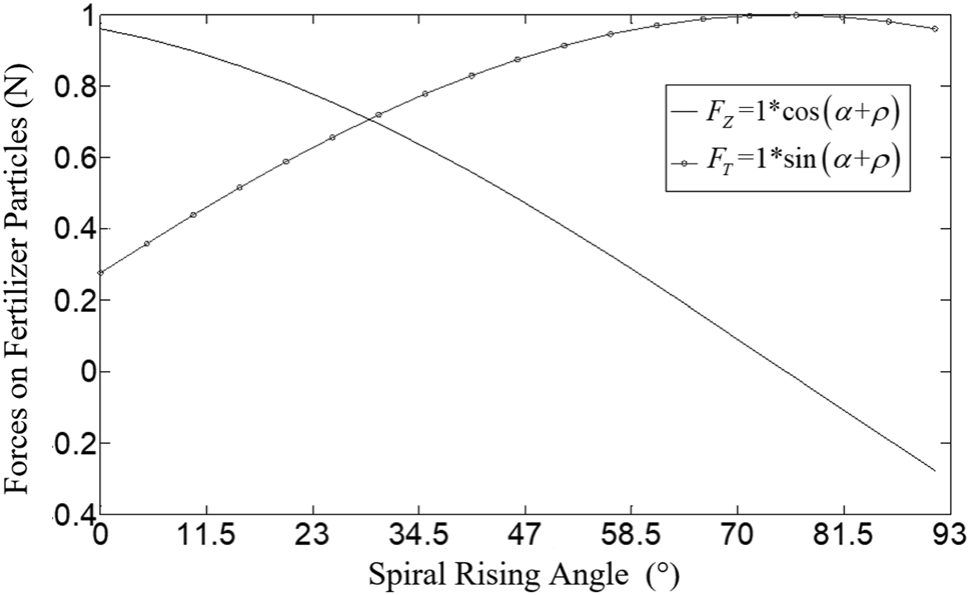
The curves of *F*_T_ and *F*_Z_ with different spiral rising angles.

According to the measured fertilizer particle size^25^ and based on the measurement principle of the inclined plane method, the friction angle of fertilizer particles is determined to be 15.74° by the self-made friction coefficient measuring instrument, as shown in Figure 6. In the case of other parameters being determined and the resultant force F fixed (assuming 1N), the curves of the circular force *F*_T_ and axial force *F*_Z_ of fertilizer particles in the spiral groove wheel with the change of the spiral rising angle are shown in Figure 7.

As shown in Figure 7, with the increase of the spiral rising angle *α*, *F*_T_ firstly increases and then decreases, reaching the maximum at 74.26°. *F*_Z_ decreases with the increase of *α*. The increase of circumferential force is beneficial to the smooth discharge of fertilizer in the spiral groove, but if it is too large which will affect the filling rate of fertilizer particles in the spiral groove, resulting in uneven fertilization. The reduction of axial force is conducive to reducing the axial movement distance of fertilizer particles in the spiral groove wheel and improving the uniformity of fertilization. However, if *α* is larger, the spiral groove wheel will be closer to the ordinary straight groove wheel, and the "pulsation phenomenon" will be more significant, which will reduce the uniformity of fertilization.

According to Figure 4 and Equation (3), when the spiral rising angle of the fertilizer wheel is 0°, the groove wheel edge is distributed along the circumference of the fertilizer wheel. This structure is not used in practical applications because the fertilizer is easy to "slip" in the groove wheel causing fertilizer blockage, which is not conducive to the discharge of fertilizer at the fertilizer outlet. When the spiral rising angle of the fertilizer wheel is 90° which means a straight groove wheel, there is only circular force but no axial force, and the fertilizer particles will not occur axial relative movement after filling the groove, which makes more fertilizer discharge at the groove and less at the groove edge, resulting in intermittent fertilizer discharge and obvious pulsation. Therefore, in combination with relevant literature^26,27^ and the above analysis, the horizontal range of the spiral rising angle is 45°–85°.

According to the working principle of the fertilizer apparatus, the groove radius, spiral rising angle, and groove wheel rotation speed are the main parameters affecting the fertilization performance. Therefore, the formulas for the fertilization amount and variation coefficient of fertilization amount are as follows:

4 $$q = \frac{1}{n}\sum\limits_{i = 1}^{n} {q_{i} } ,$$

5 $$S = \sqrt {\frac{{\sum\limits_{i = 1}^{n} {(q_{i} - q)^{2} } }}{n - 1}} ,$$

6 $$V = \frac{S}{q} \times 100,$$

where *q*_*i*_ is the fertilization amount applied to each cell, g; *q* is the average of the fertilization amount applied to each cell, g; *S* is the standard deviation of the fertilization amount applied to each cell, g; n is the number of cells; V is the variation coefficient of fertilization amount, %.

#### Parameter design of the inclined fertilizer tongue

The position and length of the fertilizer tongue ensure that will not flow automatically under the static state. The outer end of the fertilizer tongue is made into an inclined shape, which is conducive to improving the uniformity of fertilization^28^.

**Figure 8:**
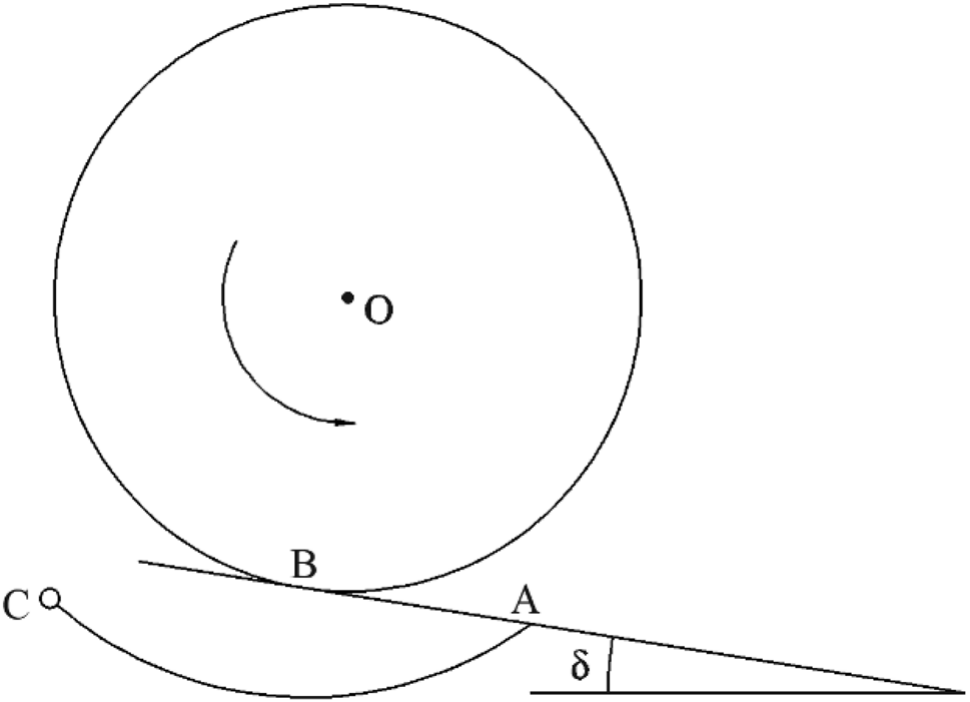
The design requirement drawing of the fertilizer tongue.

**Figure 9:**
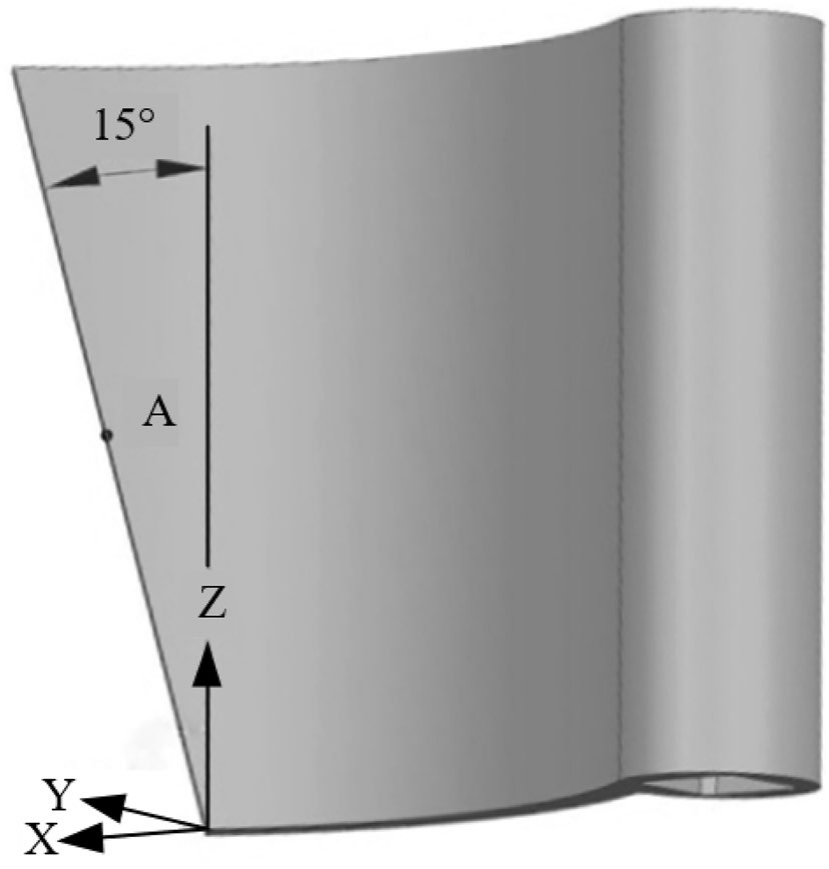
The fertilizer tongue with an inclination of 15°.

The Figure 8 shows the design requirement of the fertilizer tongue. In the figure, AC is the fertilizer tongue that can swing around C; AB is the tangent line of the outer circle O of the groove wheel; The included angle *δ* between AB and the horizontal line shall be smaller than the natural repose angle of fertilizer particles. The natural repose angle of the fertilizer particles used in the experiment is determined to be 38°. The repose angle of fertilizer particles is generally 35°– 44°, and the corresponding angle *δ* is 32.15° when the inclination angle of the fertilizer tongue is 20°^24^, which meets the design requirements. Therefore, the value range of the inclination angle of the fertilizer tongue is 5°–20°, the fertilizer tongue with an inclination of 15° is shown in Figure 9.

### Experiment and analysis

#### Experiment equipment and conditions

The traditional external groove wheel fertilizer apparatus is mainly straight groove type (the spiral rising angle is 90°), and the fertilizer is discharged at the fertilizer outlet in the form of gravity flow. When the groove wheel turns to the groove, the fertilizer in the whole groove is discharged at the same time. At this time, more fertilizer is discharged, which is called the "crest". While less fertilizer is discharged at the tooth ridge, which is called the "trough". As shown in Figure 4a, assuming that *t* is the time required for the groove wheel to rotate through the two grooves in the figure, the cycle of "crests and troughs" is carried out twice within *t* time. At this time, the fertilizer flow shows a periodic pulsation phenomenon with a pulse frequency of 2/*t*, which affects the uniformity of fertilization and is not conducive to precision fertilization, especially at low speed and small grooves. To solve the above problems, the straight groove wheel is changed to a spiral groove wheel in paper, as shown in Figure 4b. Since the groove wheel is spiral, the fertilizer is continuously discharged at the fertilizer outlet, which effectively reduces the "crests and troughs" in the pulsation phenomenon and significantly reduces the difference between the maximum and minimum values, thus improving the uniformity of fertilization.

**Figure 10:**
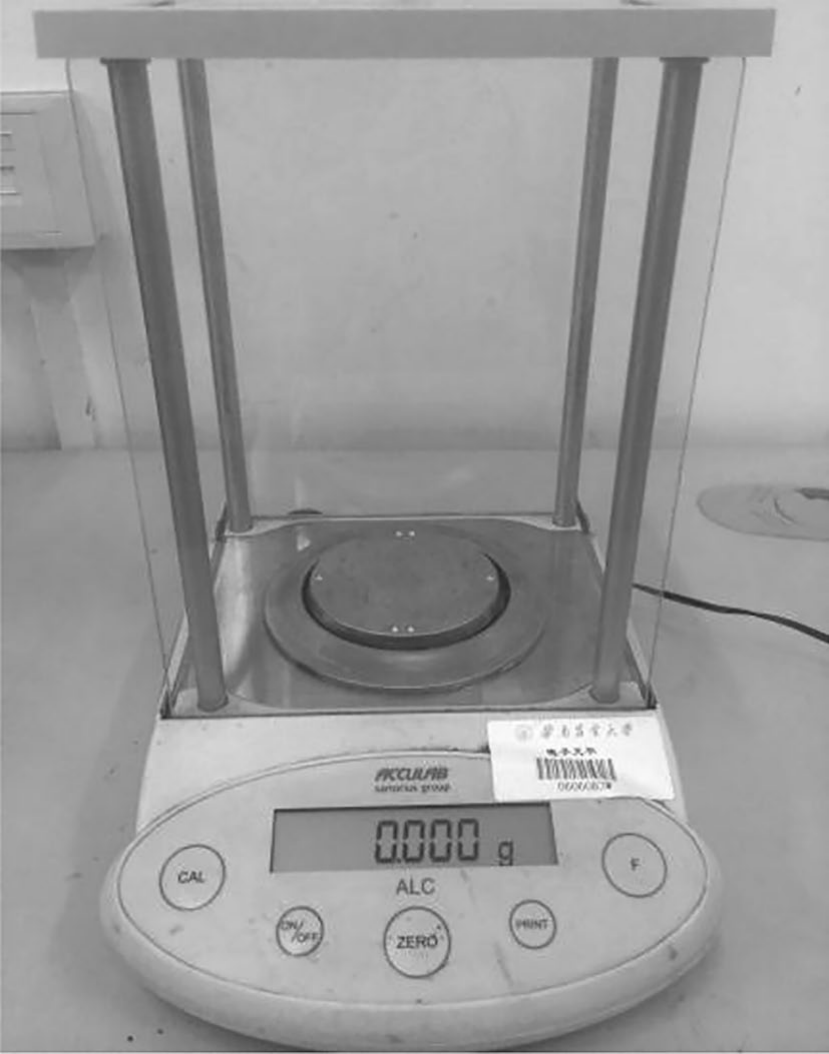
ALC-210.3 electronic balance.

**Figure 11:**
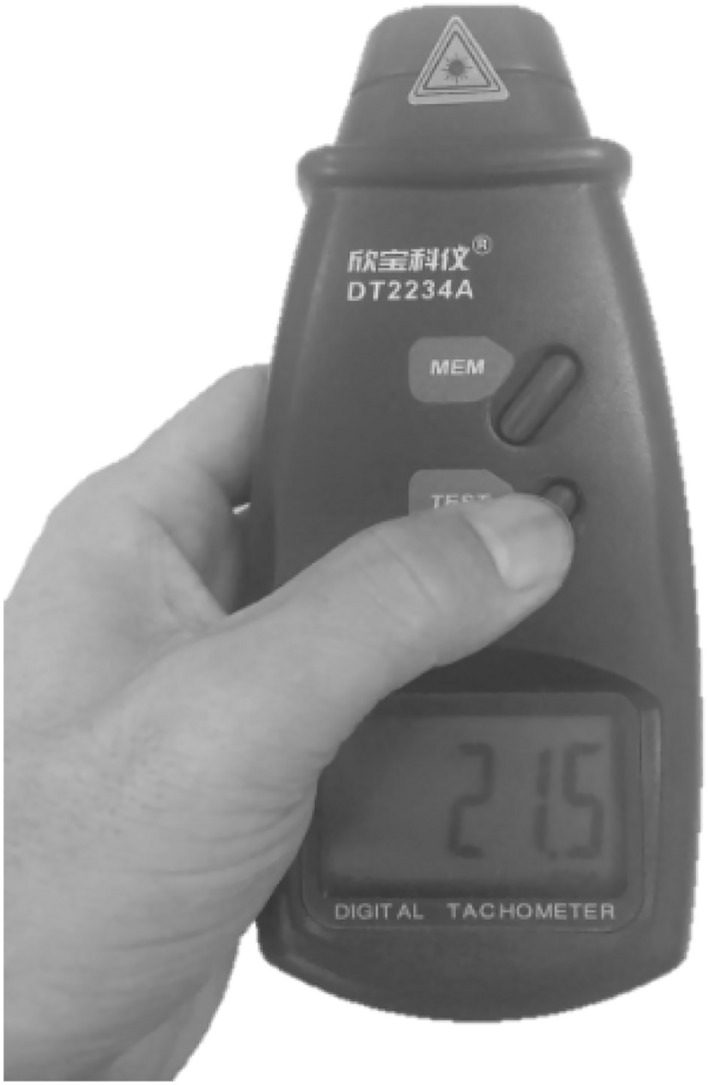
SM2234A non-contact tachometer.

The experiment materials and equipment used in this paper are listed in Table 1. The ALC-210.3 electronic balance and SM2234A non-contact tachometer are shown in Figures 10 and 11 respectively.

**Table 1:** The experiment materials and equipment used in this paper.

Name	Parameter	Manufacturer
Einstart 3D printer	The maximum printing size is 160 mm × 160 mm × 160 mm	Shining 3D Tech. Co., Ltd
ALC-210.3 electronic balance	Accuracy is 0.001 g	Sartorius Scientific Instruments (Beijing) Co., Limited
Self-made fertilizer experiment bench	/	/
DC reduction motor	12 V, 380W	Henan Yuxin Motor Company
KN-50A governor	500W	Xianxian Kenong Electronic Equipment Factory
SM2234A non-contact tachometer	Maximum measuring speed is 99,999 RPM	Shenzhen Sanpo Instrument Co., Ltd
Organic compound fertilizer	Equivalent diameter 3.24 mm; Spherical rate 97.64%	Zhongnong Jibang Fertilizer Import and Export Co., Ltd

**Figure 12:**
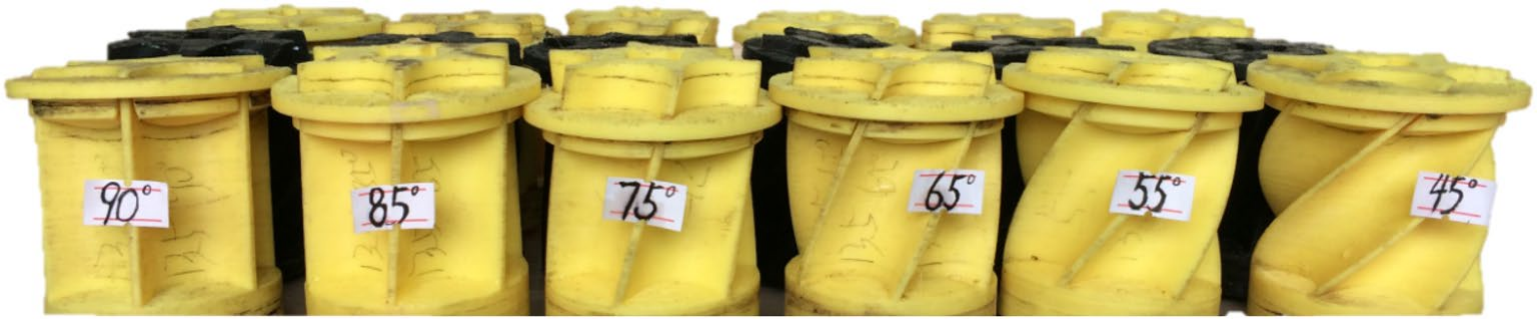
Groove wheels formed by 3D printing.

Based on the FDM type 3D printing technology, the groove wheel in the experiment and analysis is rapidly formed. As shown in Figure 12, this forming method does not require complex and expensive processes in traditional groove wheel manufacturing, which reduces the production cost and improves the experiment efficiency.

**Figure 13:**
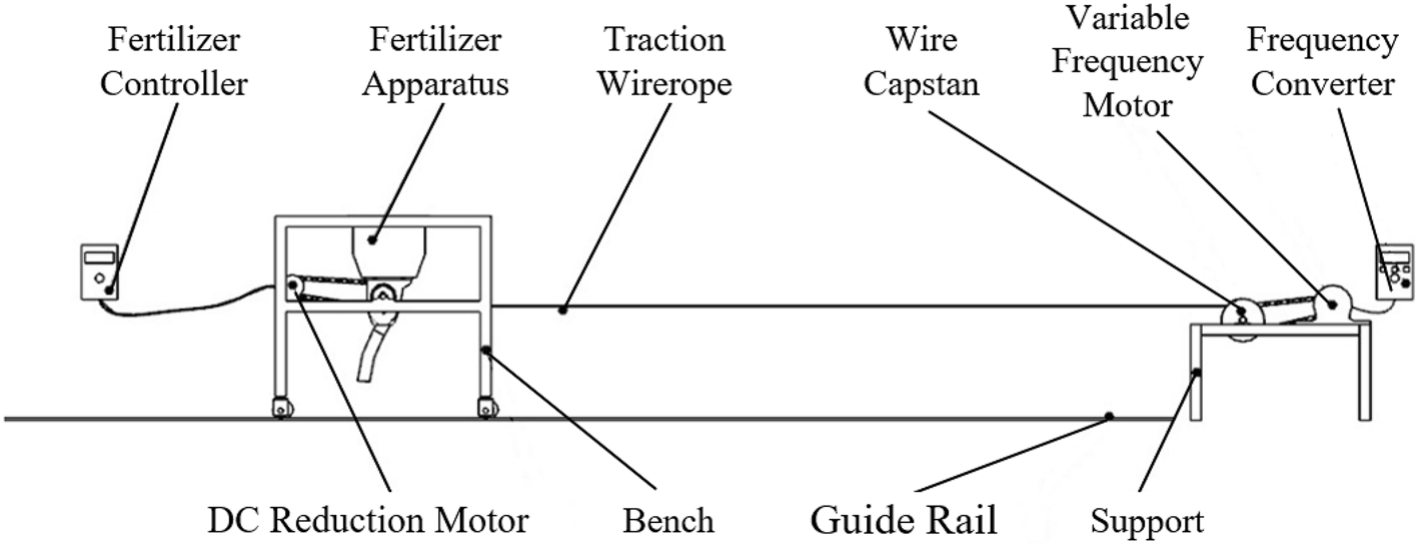
The schematic Diagram of the self-made fertilizer experiment bench.

**Figure 14:**
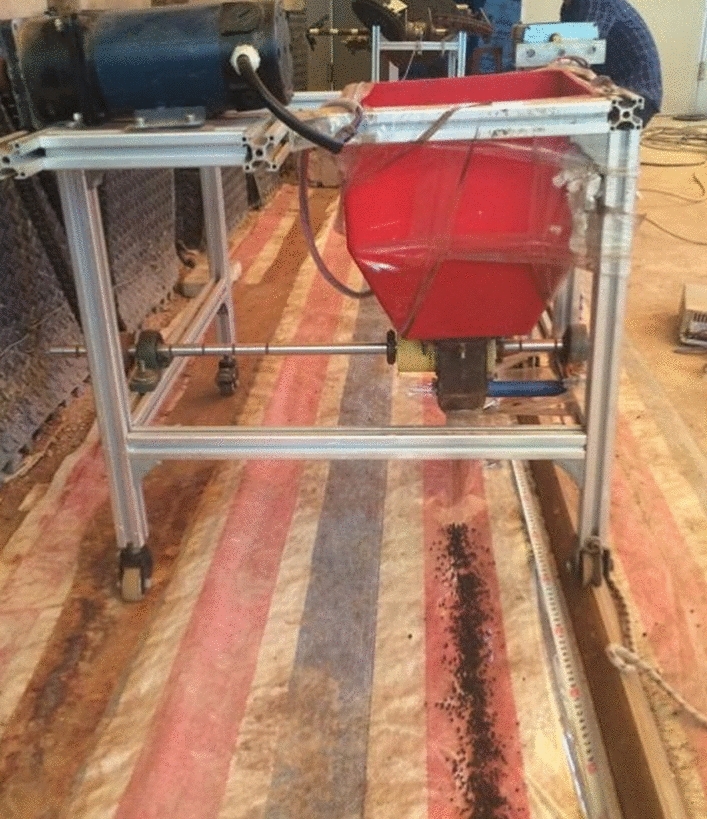
The fertilization experiment ground of the bench experiment.

The fertilization performance of the spiral groove wheel fertilizer apparatus is experimented on the self-made fertilizer experiment bench. As shown in Figure 13, the self-made fertilizer experiment bench mainly includes fertilizing parts and walking parts. The motor pulls the fertilizer bench to walk at a constant speed on the guide rail, and the fertilizer is forced to be discharged under the rotation of the groove wheel. Based on the gaps in the orchard crop plantings, the weight of the fertilizer in each unit was measured in 150mm units, which is the main amount of fertilizer received by each crop^29^. Take a distance of 150mm as a measuring unit, and measure the weight of fertilizer in each unit. Each parameter is repeated 5 times, and the arithmetic mean value is used to calculate the average fertilization amount and the variation coefficient of fertilization amount. The fertilization experiment ground of the bench experiment is shown in Figure 14. The fertilization amount tended to increase linearly with increasing groove radius during the experiment, and the variation coefficient of fertilization amount tended to decrease with increasing groove radius. The changes in the spiral rising angle have no significant impact on the fertilization amount, but have a significant impact on the coefficient of variation of the fertilization amount.

#### Experiment scheme and results

The factors that affect the fertilization performance of the fertilizer apparatus are the groove radius, spiral rising angle, groove wheel rotation speed, and fertilizer tongue inclination of the spiral groove wheel type fertilizer wheel. In this paper, the fertilization amount and the variation coefficient of fertilization amount are taken as the experiment index, the CCD (Central Composite Experimental Design) method is used to conduct the response surface analysis experiment of four factors and five levels on the above factors. Table 2 is the symbol comparison table of influencing factors and experiment indexes, and the factor codes and experiment design levels are listed in Table 3.

**Table 2:** The symbol comparison table of influencing factors and experiment indexes.

Parameter	Symbol
Groove radius	*x*_1_
Spiral rising angle	*x*_2_
Groove wheel rotation speed	*x*_3_
Fertilizer tongue inclination	*x*_4_
Fertilization amount	*y*_1_
Variation coefficient of fertilization amount	*y*_2_

**Table 3:** The factor codes and experiment design levels.

Coding	*x*_1_ (mm)	*x*_2_ (°)	*x*_3_ (r/min)	*x*_4_ (°)
2	13.5	85	35	20
1	12.5	75	30	15
0	11.5	65	25	10
-1	10.5	55	20	5
-2	9.5	45	15	0
2	13.5	85	35	20

The Design-Expert Software 10 is used to analyze the experiment data, and the regression equation is obtained by linear and quadratic polynomial fitting of the experiment data. The most appropriate model is selected according to the *F* test and *p* value to obtain the corresponding statistical results and response surface analysis results. Experiment schemes and experiment results are listed in Table 4.

**Table 4:** Experiment schemes and experiment results.

Experiment serial number	*x*_1_ (mm)	*x*_2_ (°)	*x*_3_ (r/min)	*x*_4_ (°)	*y*_1_ (g)	*y*_2_ (%)
1	10.5	55	20	5	8.309	8.98
2	12.5	55	20	5	10.940	5.38
3	10.5	75	20	5	9.051	11.53
4	12.5	75	20	5	11.192	5.84
5	10.5	55	30	5	12.286	6.31
6	12.5	55	30	5	16.818	5.14
7	10.5	75	30	5	13.134	5.72
8	12.5	75	30	5	16.606	6.33
9	10.5	55	20	15	8.488	6.76
10	12.5	55	20	15	11.817	5.95
11	10.5	75	20	15	9.356	8.29
12	12.5	75	20	15	12.206	6.59
13	10.5	55	30	15	12.996	4.95
14	12.5	55	30	15	17.691	6.50
15	10.5	75	30	15	13.562	6.77
16	12.5	75	30	15	17.776	5.64
17	9.5	65	25	10	10.568	9.28
18	13.5	65	25	10	15.794	5.26
19	11.5	45	25	10	12.404	6.33
20	11.5	85	25	10	13.277	8.35
21	11.5	65	15	10	8.132	10.83
22	11.5	65	35	10	18.797	6.78
23	11.5	65	25	0	13.296	5.85
24	11.5	65	25	20	15.380	5.50
25	11.5	65	25	10	13.338	5.01
26	11.5	65	25	10	13.253	4.83
27	11.5	65	25	10	13.586	5.26
28	11.5	65	25	10	13.211	6.73
29	11.5	65	25	10	13.009	5.52
30	11.5	65	25	10	13.887	5.08
31	11.5	65	25	10	13.255	5.97
32	11.5	65	25	10	13.829	5.09
33	11.5	65	25	10	13.489	5.55
34	11.5	65	25	10	13.420	5.41
35	11.5	65	25	10	13.115	6.29
36	11.5	65	25	10	13.114	5.25

#### Variance analysis of the experiment results

The stepwise regression method is adopted to eliminate insignificant factors, and the quadratic regression equation variance analysis is conducted for the fertilization amount and the variation coefficient of fertilization amount respectively. The analysis results are listed in Table 5 and 6 respectively. The results of variance analysis show that the two models have extremely significant (*P*<0.01), the fitting coefficients are 0.9651 and 0.8389 respectively. The error is mainly caused by non-uniform fertilizer particles and a small part of the particles broken during fertilization. The RSM (Response Surface Method) model can generally be considered effective if its fitting coefficient exceeds 0.80. Therefore, the fitting degree of the two models is high, which has certain practical significance.

**Table 5:** The variance analysis of the fertilization amount.

Sources of variance	Sum of squares	Degrees of freedom	The mean square	*F*	*P*
Model	226.15	7	32.31	121.15	< 0.0001
*x*_1_	61.17	1	61.17	229.4	< 0.0001
*x*_2_	1.16	1	1.16	4.36	0.0510
*x*_3_	154.23	1	154.23	578.37	< 0.0001
*x*_4_	3.94	1	3.94	14.77	0.0008
*x*_1_*x*_3_	2.22	1	2.22	8.33	0.0087
*x*_2_^2^	2.33	1	2.33	8.74	0.0100
Residual	7.47	28	0.27		
Unfit term	7.57	18	0.42	8.13	0.0005
Pure error	6.61	17	0.39	5.01	
Total dispersion	0.85	11	0.078		
*R*^2^ = 0.9651

**Table 6:** The variance analysis of the variation coefficient of fertilization amount.

Sources of variance	Sum of squares	Degrees of freedom	The mean square	*F*	*P*
Model	75.8	9	8.42	15.04	< 0.0001
*x*_1_	16.63	1	16.63	29.71	< 0.0001
*x*_2_	4.84	1	4.84	8.65	0.0068
*x*_3_	16.77	1	16.77	29.95	< 0.0001
*x*_4_	0.84	1	0.84	1.49	0.2326
*x*_1_*x*_3_	8.5	1	8.5	15.18	0.0006
*x*_1_*x*_4_	3.76	1	3.76	6.72	0.0154
*x*_1_^2^	3.68	1	3.68	6.57	0.0165
*x*_2_^2^	4.07	1	4.07	7.27	0.0122
*x*_3_^2^	16.72	1	16.72	29.86	< 0.0001
Residual	14.56	26	0.56		
Unfit term	11.12	15	0.74	2.38	0.0764
Pure error	3.43	11	0.31		
Total dispersion	90.36	35			
*R*^2^ = 08,389					

As shown in Table 5, according to the *F* value of each factor, within the selected factor level range, the groove wheel rotation speed (*x*_3_) is the most influential factor on the fertilization amount, and after that the groove radius (*x*_1_), the fertilizer tongue inclination (*x*_4_), the quadratic effect of the spiral rising angle (*x*_2_^2^), the interaction between the groove radius and the groove wheel rotation speed (*x*_1_*x*_3_) and the spiral rising angle (*x*_2_).

Similarly, Table 6 shows that the groove wheel rotation speed (*x*_3_) is the most influential factor on the variation coefficient of fertilization amount, and after that the quadratic effect of the groove wheel rotation speed (*x*_3_^2^), the groove radius (*x*_1_), the interaction between the groove radius and the groove wheel rotation speed (*x*_1_*x*_3_), the spiral rising angle (*x*_2_), the quadratic effect of the spiral rising angle (*x*_2_^2^), the interaction between the groove radius and the fertilizer tongue inclination (*x*_1_*x*_4_), the quadratic effect of the groove radius (*x*_1_^2^) and the fertilizer tongue inclination (*x*_4_).

Based on the experimental results and analysis results, the fitting mathematical models of fertilization amount and the variation coefficient of fertilization amount are obtained as follows:

7 $$y_{1} = 13.22 + 1.6x_{1} + 0.22x_{2} + 2.53x_{3} + 0.41x_{4} + 0.37x_{1} x_{3} - 0.26x_{2}^{2}$$

8 $$y_{2} = 5.47 - 0.83x_{1} + 0.45x_{2} - 0.84x_{3} - 0.19x_{4} + 0.73x_{1} x_{3} + 0.48x_{1} x_{4} + 0.34x_{1}^{2} + 0.36x_{2}^{2} + 0.72x_{3}^{2}$$

#### Response surface analysis

**Figure 15:**
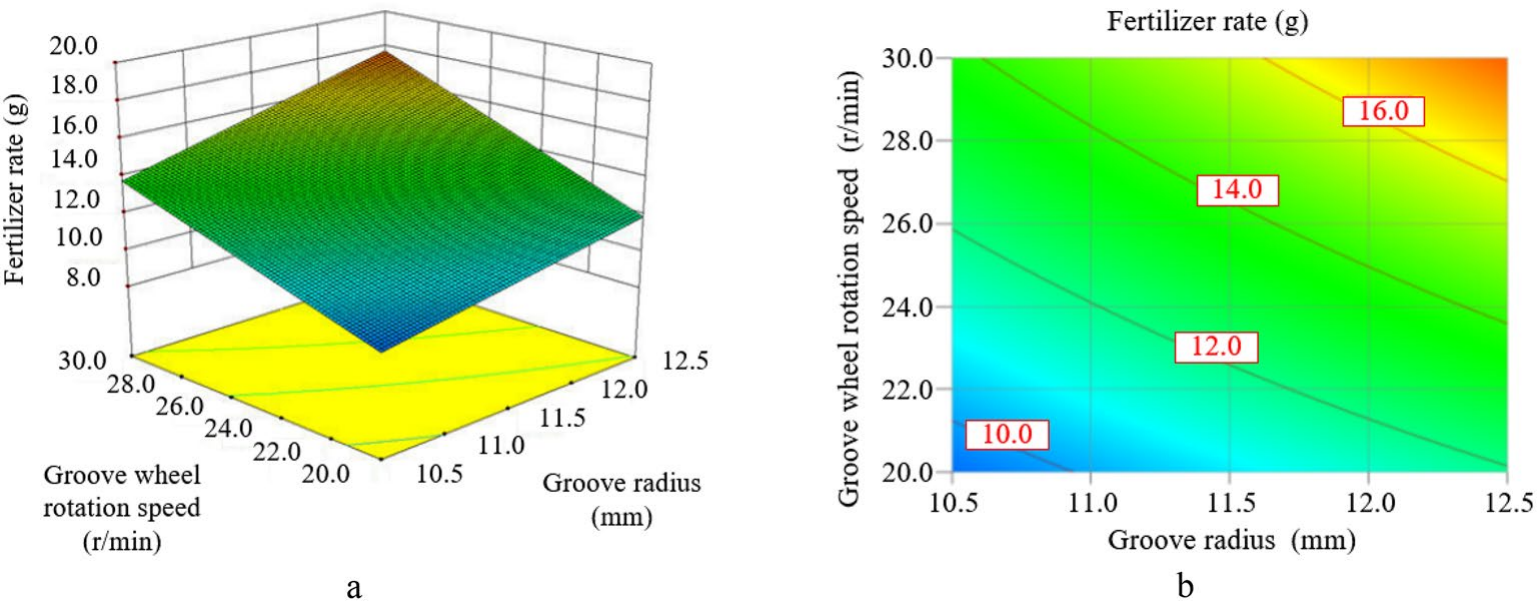
The interaction between the groove radius and the groove wheel rotation speed on the fertilization amount.

The Figure 15 shows the response diagram and contour map of the influence of the groove radius and the groove wheel rotation speed on the fertilization amount when the spiral rising angle is 65° and the fertilizer tongue inclination is 10°. As shown in Figure 15, the interaction between the groove radius and the groove wheel rotation speed has a significant impact on the fertilization amount. The fertilization amount increases with the increase in the groove radius and the groove wheel rotation speed, and the increasing trend caused by the groove wheel rotation speed is more significant than that caused by the groove radius. When the groove radius and the groove wheel rotation speed increase at the same time, and close to the limit point (12.5 mm, 30 r/min) in the figure, the increase trend of the curve surface in the response diagram for the fertilization amount is more significant. In addition, when the spiral rising angle changes within the selected value range, the volume change in the groove of the groove wheel is less than 0.1% due to the same cross-sectional area of the groove wheel. So the fertilizer filling amount is basically the same and the spiral rising angle has little impact on the fertilization amount.

**Figure 16:**
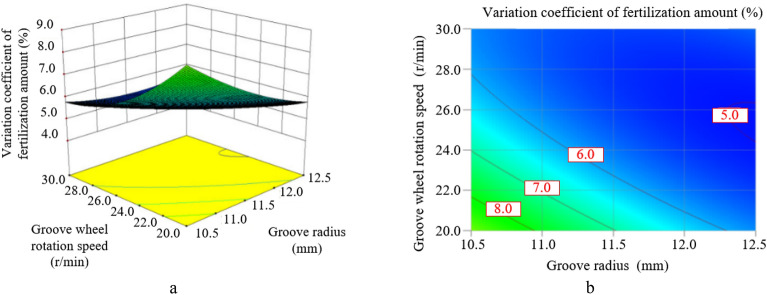
The interaction between the groove radius and the groove wheel rotation speed on the variation coefficient of fertilization amount.

The Figure 16 shows the response diagram and contour map of the influence of the groove radius and the groove wheel rotation speed on the variation coefficient of fertilization amount when the spiral rising angle is 65° and the fertilizer tongue inclination is 10°. As shown in Figure 16, the groove radius and the groove wheel rotation speed have an interactive effect on the variation coefficient of fertilization amount. When the groove wheel rotation speed is less than 25 r/min, the variation coefficient of fertilization amount decreases with the increase of the groove radius, and the variation trend is particularly significant at low rotational speed and shows a linear correlation. When the groove wheel rotation speed exceeds 25 r/min, the influence of the groove radius on the variation coefficient of fertilization amount decreases gradually, no longer showing linear correlation, but showing a trend of decreasing first and then increasing with the increase of the groove radius. Similarly, the influence of the groove wheel rotation speed on the variation coefficient of fertilization amount is basically the same as that of the groove radius. When the groove radius is less than 11.5 mm, the variation coefficient of fertilization amount can be increased by reducing the groove wheel rotation speed. When the groove radius is more than 11.5 mm, the influence of the groove wheel rotation speed on the variation coefficient of fertilization gradually decreases, and even appears a trend of first decreasing and then increasing. On the whole, when the groove radius and the groove wheel rotation speed increase at the same time, the variation coefficient of fertilization amount decreases first and then increases.

**Figure 17:**
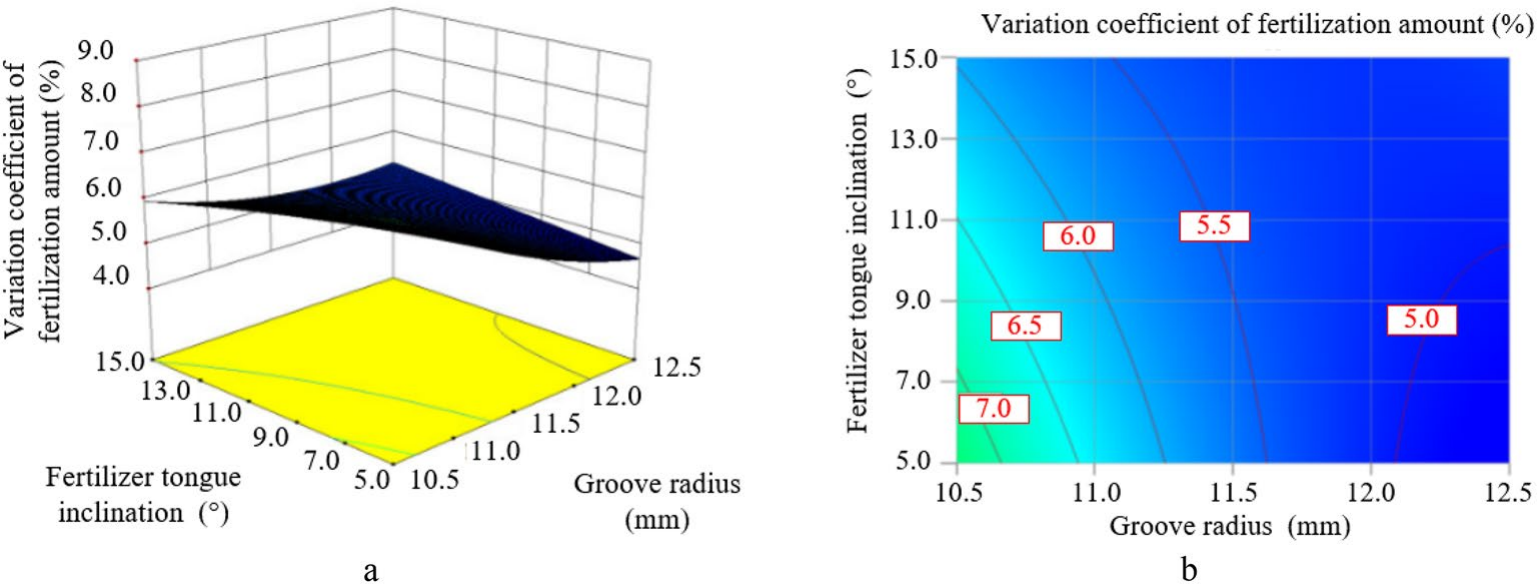
The interaction between the fertilizer tongue inclination and the groove radius on the variation coefficient of fertilization amount.

The Figure 17 shows the response diagram and contour map of the influence of the fertilizer tongue inclination and the groove radius on the variation coefficient of fertilization amount when the spiral rising angle is 65° and the groove wheel rotation speed is 25 r/min.

As shown in Figure 17, the interaction between the fertilizer tongue inclination and the groove radius has a significant impact on the variation coefficient of fertilization amount. When the fertilizer tongue inclination is constant, the variation coefficient of fertilization amount increases with the decrease in the groove radius, and the changing trend under the small fertilizer tongue inclination is more obvious than that under the large fertilizer tongue inclination. When the groove radius is smaller than the central level (11.5 mm), the variation coefficient of fertilization amount can be decreased by increasing the fertilizer tongue inclination. When the groove radius is larger than the central level, the variation coefficient of fertilization amount increases with the increase of the fertilizer tongue inclination. Taken overall, when the fertilizer tongue inclination and the groove radius decrease at the same time, the variation coefficient of fertilization amount shows a trend of increasing first and then decreasing. Furthermore, when the small fertilizer tongue inclination and the large groove radius are combined, the variation coefficient of fertilization amount is relatively small.

#### Optimization design and validation

To obtain the best parameter combination, a multi-objective optimization method is adopted. The objective function *y*_1_ and *y*_2_ are Equations (7) and (8) respectively, and the optimization equation is in the form of:

9 $$\left\{ \begin{gathered} 9.5 \le x_{1} \le 13.5 \hfill \\ 45 \le x_{2} \le 85 \hfill \\ 15 \le x_{3} \le 30 \hfill \\ 5 \le x_{4} \le 15 \hfill \\ y_{1} = f_{1} (x_{1} ,x_{2} ,x_{3} ,x_{4} ) \hfill \\ y_{2} = f_{2} (x_{1} ,x_{2} ,x_{3} ,x_{4} ) \hfill \\ \max y_{1} \hfill \\ \min y_{2} \hfill \\ \end{gathered} \right.$$

Based on the above optimization conditions, the predicted and actual optimization results are listed in Table 7. Under the optimized conditions, the error between the predicted value of the fertilization amount and the actual value is 4.11%, and the error between the predicted value of the variation coefficient of fertilization amount and the actual value is 5.51%, both of which are within a reasonable range, indicating the reliability of the optimization results^29^.

**Table 7:** The predicted and actual optimization results under optimization conditions.

Optimal solution	Predicted optimization results	Actual optimization results
Groove radius (mm)	13.5	13.5
Spiral rising angle (°)	61.85	62
Groove wheel rotation speed (r/min)	29.38	29.4
Fertilizer tongue inclination (°)	5	5
Fertilization amount (g)	18.792	17.810
Variation coefficient of fertilization amount (%)	5.37	5.60

**Figure 18:**
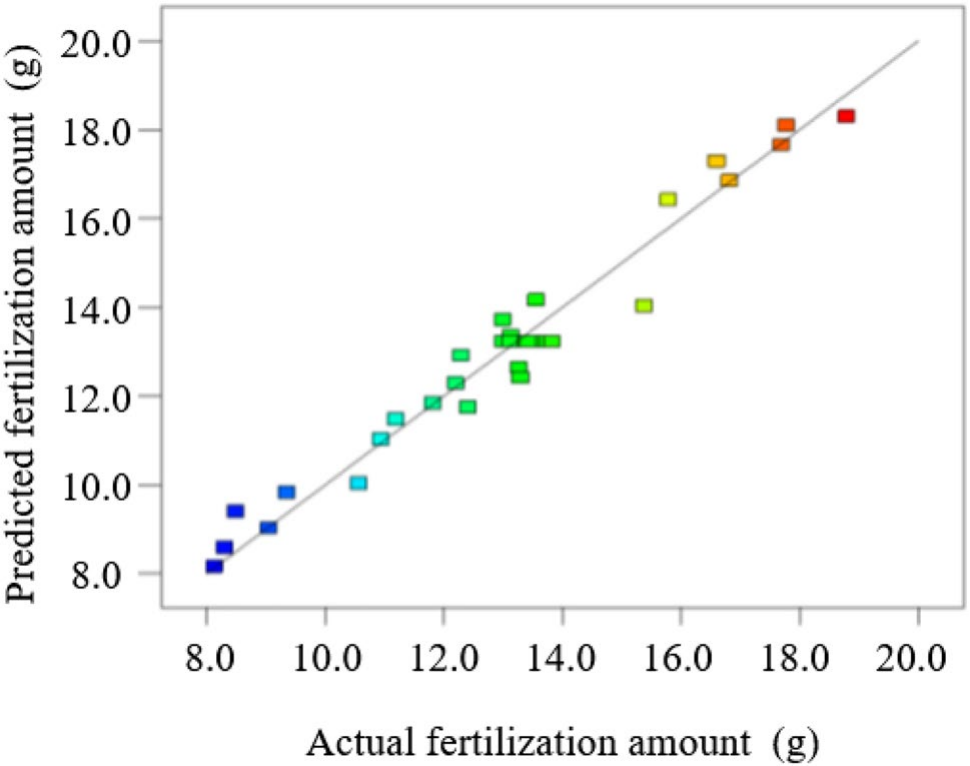
Scattered distribution of the fertilization amount.

**Figure 19:**
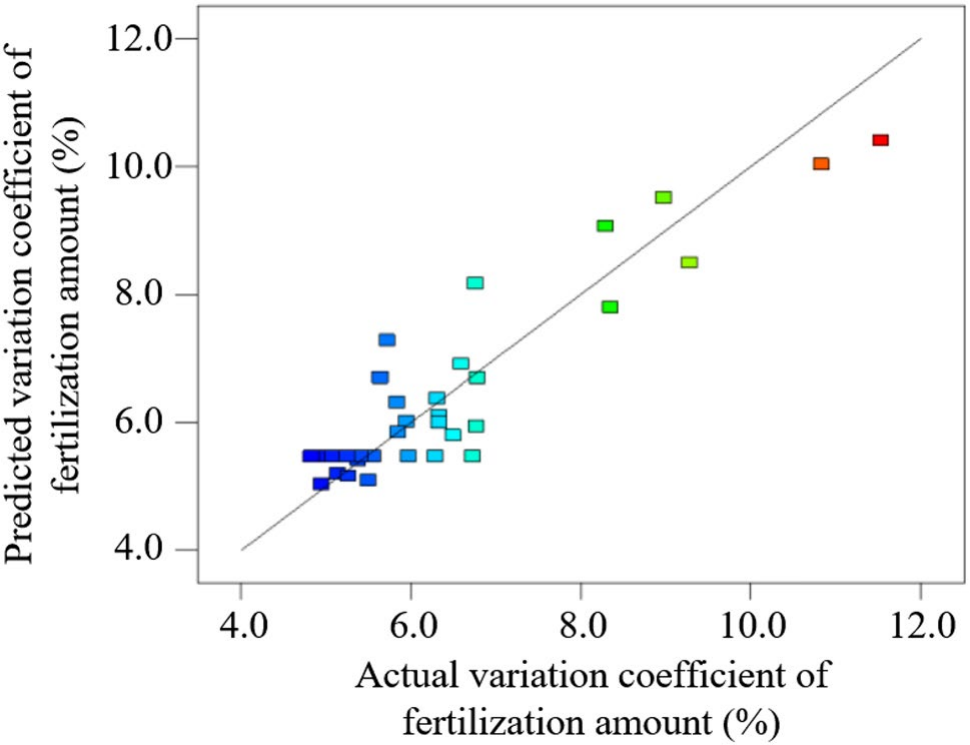
Scattered distribution of the variation coefficient of fertilization amount.

For further verifying the accuracy and reliability of the established quadratic regression model, the self-made fertilizer experiment bench is used for test validation within the experimental range. The relationship between the measured value and the predicted value of the regression model of the fertilization amount and the variation coefficient of fertilization amount are shown in Figures 18 and 19 respectively. The predicted value and the actual value are generally distributed diagonally, indicating that the predicted value of the model is consistent with the actual measured value.

## Conclusion

To solve the problem that the fertilizer flow fluctuates periodically and is not conducive to precision fertilization of the commonly used straight-grooves type external groove wheel fertilizer apparatus, a kind of the spiral groove wheel fertilizer apparatus is proposed in the paper. By changing the straight groove wheel to the spiral groove wheel, the fertilizer is continuously discharged at the fertilizer outlet, effectively reducing the "crests and troughs" in the pulsation phenomenon and improving the uniformity of fertilization. The structure of the spiral groove wheel fertilizer apparatus and the dynamic characteristics of fertilizer particles in the fertilization are analyzed, and the interaction effects of various structural parameters on fertilization performance are discussed by using Design-Expert Software 10 and a self-made fertilizer experiment bench. The optimal size of the spiral groove wheel fertilizer apparatus is determined through optimization and experimental verification. The detailed conclusions are as follows:

1. Within the selected factor level range, the interaction between the groove radius and the groove wheel rotation speed has a significant impact on the fertilization amount. The fertilization amount increases with the increase in the groove radius and the groove wheel rotation speed, and the increasing trend caused by the groove wheel rotation speed is more significant than that caused by the groove radius. In addition, the groove wheel rotation speed is the most influential factor on the fertilization amount, and after that the groove radius, the fertilizer tongue inclination, the quadratic effect of the spiral rising angle, the interaction between the groove radius and the groove wheel rotation speed and the spiral rising angle.
2. Within the selected factor level range, the groove radius, the groove wheel rotation speed, and the fertilizer tongue inclination have interactive effects on the variation coefficient of fertilization amount. The variation coefficient of fertilization amount increases with the decrease in the groove radius under a constant fertilizer tongue inclination. When the groove radius and the groove wheel rotation speed are less than the central level, which the groove radius is 11.5 mm and the groove wheel rotation speed is 25 r/min, the variation coefficient of fertilization amount can be decreased by increasing the groove radius, the groove wheel rotation speed and the fertilizer tongue inclination. When the groove radius and the groove wheel rotation speed are larger than the central level, the variation coefficient of fertilization amount decreases first and then increases with the increase of the groove radius and the groove wheel rotation speed, and increases with the increase of the fertilizer tongue inclination. Furthermore, the groove wheel rotation speed is the most influential factor on the variation coefficient of fertilization amount, and after that the quadratic effect of the groove wheel rotation speed, the groove radius, the interaction between the groove radius and the groove wheel rotation speed, the spiral rising angle, the quadratic effect of the spiral rising angle, the interaction between the groove radius and the fertilizer tongue inclination, the quadratic effect of the groove radius and the fertilizer tongue inclination.
3. Combined with the experimental validation, the response surface method and multi-objective optimization algorithm are adopted to optimize the design. The final parameter set of the spiral groove wheel fertilizer apparatus is: the groove radius is 13.5 mm, the spiral rising angle is 62°, the groove wheel rotation speed is 29.4r/min and the fertilizer tongue inclination is 5°, with the fertilization amount of 17.810 g and the variation coefficient of fertilization amount of 5.60%.
4. The quadratic regression models of multiple factors are established for the fertilization amount and the variation coefficient of fertilization amount with regard to the design variables. The accuracy and reliability of the regression models are verified by using the self-made fertilizer experiment bench. The experiment results show that the predicted value and the actual value are distributed diagonally, indicating that the predicted value of the model is consistent with the actual measured value.

## Data Availability

All data generated or analyzed during this study are included in this published article. Request for more details to the corresponding author.

## References

[1] Xiongfei C, Xiwen L, Zaiman W (2015). Design and experiment of fertilizer distribution apparatus with double-level screws. Trans. Chin. Soc. Agric. Mach..

[2] Junyan Y (2016). Design of a new type of fertilizer applicator. J. Mach. Des..

[3] Dianyun C, Hongwei W, Yang F (2017). Effects of different fertilization methods and planting densities on yield and nitrogen utilization of hybrid maize Shen Nong T19. Jiangsu Agric. Sci..

[4] Hao L (2014). A New Kind of Method for the Optimized Design of Outer Groove-Wheel Fertilizer Apparatuses.

[5] Fountas S, Blackmore S, Ess D (2015). Farmer experience with precision agriculture in Denmark and the US eastern corn belt. Precis. Agric..

[6] Laurent BFC, Bridgwater J (2002). Dispersive granular flow in a horizontal drum stirred by a single blade. AICHE J..

[7] Van Liedekerke P, Tijskens E, Dintwa E (2006). A discrete element model for simulation of a spinning disc fertilizer spreader I. Single Part. Simul. Powder Technol..

[8] Youlu B (2016). Analysis of the development and the demands of fertilization machinery. Soil Fertil. Sci. China.

[9] Peng L (2013). Theoretical and Experimental Study on Fertilizer Spreading Machines of Swing Tube Type.

[10] Guoqiang D, Haitao C, Yining F (2016). Parameter optimization and test of key parts of fertilizer allocation device based on EDEM software. Trans. Chin. Soc. Agric. Eng..

[11] Xiong G, Lei H (2016). Study on fertilizer experiment of 2CMG-4 type potato planter. J. Agric. Mech. Res..

[12] Wei Z, Fulin W, Chun W (2005). Experimental study on 2BD-7type multifunctional precision seed spacing drill. Trans. Chin. Soc. Agric. Eng..

[13] Yuxue G, Jin Y, Chengliang L (2011). FIS-based method to generate bivariate control parameters regulation sequence for fertilization. Trans. Chin. Soc. Agric. Eng..

[14] Xiu W, Chunjiang Z, Zhijun M (2004). Design and experiment of variable rate fertilizer applicator. Trans. Chin. Soc. Agric. Eng..

[15] Guoming H (2010). Analytical Simulation of Granular Systems by Discrete Element Method.

[16] Zheng M, Yaoming L, Lizhang X (2013). Summarize of particle movements research in agricultural engineering realm. Trans. Chin. Soc. Agric. Mach..

[17] Tao C, Jia L, Li Y (2013). Experiment and simulation of rolling friction characteristic of corn seed based on high-speed photography. Trans. Chin. Soc. Agric. Eng..

[18] Wanyi L, Pan Z, Liping L (2012). Simulation based on discrete element method and experiment on flow and packing behavior of particles. Mech. Eng..

[19] Yanlong H, Fuguo J, Yurong T (2014). Influence of granular co efficient of rolling friction on accumulation characteristics. Acta Phys. Sin-Ch Ed..

[20] Ning S (2016). Research on Variable Rate Fertilization Control Technology in Precision Agriculture.

[21] Ruru X, Ruicheng D, Huawei L (2010). Design on double-hole former helm-shaped pneumatic precision hill planter of seed and fertilizer. J. Agric. Mech. Res..

[22] Shizhe C, Lixin Z, Zhen L (2016). Design of double variable control system of fertilization. Xinjiang Agric. Mech..

[23] Shiqiang P, Yaxiang Z, Liang J (2016). Design and experimental research of external groove wheel fertilizer apparatus of 2BFJ-6 type variable rate fertilizer applicator. J. Chin. Agric. Mech..

[24] Chinese Academy of Agricultural Mechanization Sciences (2007). Agricultural Machinery Design Manual.

[25] Zhou Y, Qingchuang Z, Jianfeng S (2018). Study on the performance of fluted roller fertilizer distributor based on EDEM and 3D printing. J. Agric. Mech. Res..

[26] Xu M, Jianxia K, Long Q (2015). Design and experiment of precision seeder for rice paddy field seedling. Trans. Chin. Soc. Agric. Mach..

[27] Liquan T, Jinwu W, Han D (2016). Design and performance experiment of helix grooved rice seeding device. Trans. Chin. Soc. Agric. Mach..

[28] Yongmei W, Wanzhang Y, Xigui W (2006). Study on row sowing device with spiral sheave. J. Xinjiang Agric. Univ..

[29] Jiahua Z (2015). Simulation Analysis of Variable Fertilizer Applicator Apparatus Fat Process Based on Discrete Element Method.

